# Antiviral functionalization of cellulose using tannic acid and tannin-rich extracts

**DOI:** 10.3389/fmicb.2023.1287167

**Published:** 2023-12-06

**Authors:** Marjo Haapakoski, Aleksei Emelianov, Dhanik Reshamwala, Mira Laajala, Jenni Tienaho, Petri Kilpeläinen, Jaana Liimatainen, Tuula Jyske, Mika Pettersson, Varpu Marjomäki

**Affiliations:** ^1^Department of Biological and Environmental Sciences/Nanoscience Center, University of Jyväskylä, Jyväskylä, Finland; ^2^Department of Chemistry/Nanoscience Center, University of Jyväskylä, Jyväskylä, Finland; ^3^Production Systems Unit, Natural Resources Institute Finland (Luke), Helsinki, Finland

**Keywords:** antiviral functionalization, enteroviruses, coronaviruses, tannic acid, cellulose, bark extract, Raman spectroscopy

## Abstract

Due to seasonally appearing viruses and several outbreaks and present pandemic, we are surrounded by viruses in our everyday life. In order to reduce viral transmission, functionalized surfaces that inactivate viruses are in large demand. Here the endeavor was to functionalize cellulose-based materials with tannic acid (TA) and tannin-rich extracts by using different binding polymers to prevent viral infectivity of both non-enveloped coxsackievirus B3 (CVB3) and enveloped human coronavirus OC43 (HCoV-OC43). Direct antiviral efficacy of TA and spruce bark extract in solution was measured: EC_50_ for CVB3 was 0.12 and 8.41 μg/ml and for HCoV-OC43, 78.16 and 95.49 μg/ml, respectively. TA also led to an excellent 5.8- to 7-log reduction of severe acute respiratory syndrome coronavirus 2 (SARS-CoV-2) virus infectivity. TA functionalized materials reduced infectivity already after 5-min treatment at room temperature. All the tested methods to bind TA showed efficacy on paperboard with 0.1 to 1% (w/v) TA concentrations against CVB3 whereas material hydrophobicity decreased activities. Specific signatures for TA and HCoV-OC43 were discovered by Raman spectroscopy and showed clear co-localization on the material. qPCR study suggested efficient binding of CVB3 to the TA functionalized cellulose whereas HCoV-OC43 was flushed out from the surfaces more readily. In conclusion, the produced TA-materials showed efficient and broadly acting antiviral efficacy. Additionally, the co-localization of TA and HCoV-OC43 and strong binding of CVB3 to the functionalized cellulose demonstrates an interaction with the surfaces. The produced antiviral surfaces thus show promise for future use to increase biosafety and biosecurity by reducing pathogen persistence.

## Graphical abstract

A schematic presenting the antiviral functionalization of cellulose with tannic acid and tannin-rich extracts.

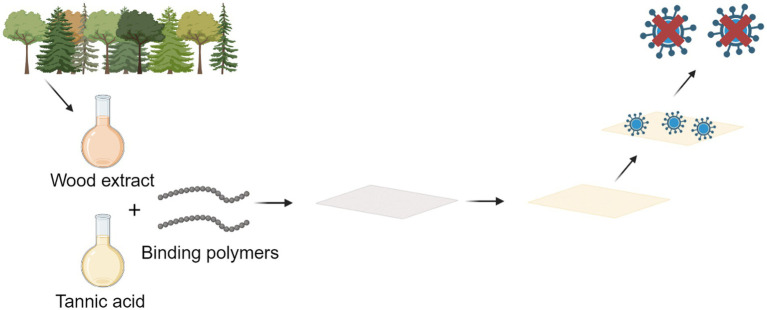

## 1 Introduction

We have recently faced coronavirus pandemic but are also regularly attacked by seasonally prevalent viruses like enteroviruses and influenza. Viruses causing respiratory tract infections are known to be transmitted via aerosols. However, several viruses can remain infectious on top of surfaces for several hours and days. For instance, infectious severe acute respiratory syndrome coronavirus 2 (SARS-CoV-2) has been detected up to 72 h on top of plastic and stainless steel, 24 h on cardboard and 4 h on copper (van Doremalen et al., 2020). A recent survey went through 78 well-controlled studies where different physical measures to stop or slow the spread of respiratory viruses were compared (Jefferson et al., 2023). The results displayed that hand hygiene is more effective than masks to prevent infections. Thus, fomites on surfaces and hands are an important pathway for viruses to infect new hosts. Disinfectants are one solution to prevent fomite transmission, but they are rarely environmentally friendly, and some are considered as irritants causing skin and/or bronchial irritation (Goh et al., 2021; Bhat et al., 2022). Disinfectants can also damage treated surfaces and constant surface cleaning is also time consuming.

Viruses are simple entities but come in different features concerning their structure and persistence. Enveloped viruses are more vulnerable to lose their infectivity due to the lipid coating whereas non-enveloped viruses including enteroviruses, tightly packed with a protein shell, are much more difficult to combat in our everyday life (Abad et al., 1994; Firquet et al., 2015; van Doremalen et al., 2020). Enteroviruses are good examples of small non-enveloped viruses: they are small, approximately 30 nm in diameter, and have a single stranded RNA genome enclosed in icosahedral capsid structure (Marjomäki et al., 2015). Enterovirus infections are very common and cause most of the common cold infections on a yearly basis. However, they can also lead to severe acute infections in secondary infection sites like pancreas, heart and the brain, causing myocarditis, pancreatitis and meningitis, respectively (Nikonov et al., 2017). Furthermore, enterovirus infections are contributing to chronic conditions like of type 1 diabetes (Oikarinen et al., 2012; Laitinen et al., 2014). Coxsackie B viruses belonging to enteroviruses are known to be able induce myocarditis and pancreatitis (Cooper, 2009; Lerch and Gorelick, 2013). Coxsackie B viruses are also relatively stable on inanimate surfaces, thus posing a risk for fomite transmission. Infectious coxsackievirus B4 (CVB4) has been detected on a petri dish for 5 weeks (Firquet et al., 2015). While human coronaviruses, such as the SARS-CoV-2 causing COVID-19 and the seasonally infecting human coronavirus OC43 (HCoV-OC43), are also single stranded RNA viruses, they are much larger compared to enteroviruses; approximately 120–160 nm in diameter. Coronaviruses have a lipid bilayer envelope that is covered by spikes. In addition to spike (S) protein, coronaviruses have 3 other structural proteins: membrane (M) protein, envelope (E) protein and nucleocapsid (N) protein (Wang M.-Y. et al., 2020; Marjomäki et al., 2021). Both enteroviruses and coronaviruses are known to transmit via respiratory tract and fecal-oral route, but fecal-oral route is even more common for enteroviruses (Wells and Coyne, 2019; Shereen et al., 2020). Coronaviruses like SARS-CoV-2 and Middle East respiratory syndrome-related coronavirus (MERS-CoV) are causing outbreaks with severe consequences (Alnuqaydan et al., 2021; Lippi et al., 2023).

Several surfaces with antimicrobial properties and coatings have been already invented and examined, but so far sustainable, safe, and natural solutions are still limited. Many currently available antiviral surfaces and coatings rely regularly on metals such as copper (Cu), silver (Ag), gold (Au), and zinc (Zn), (Rakowska et al., 2021). Potential of polymers, like synthetic polyethylenimines (PEI), dendrimers and natural chitosan (CS), hydrogels and antimicrobial peptides for antiviral purposes have also been assessed (Rakowska et al., 2021; Bregnocchi et al., 2022). In addition, there are recent approaches to use nature-based compounds to combat viral infection on various surfaces based on the shown antiviral activity (Fabra et al., 2016; Randazzo et al., 2018; Amankwaah et al., 2020; Ordon et al., 2021). Although several natural extracts and molecules possess antiviral capability, there are obvious challenges to retain the antiviral functionality on the surfaces developed. In addition, the antiviral activity may remain specific to a small number of viruses, e.g., enveloped viruses, leaving the more stubborn non-enveloped viruses still active.

TA has been associated with versatile bioactive potential (Kaczmarek, 2020). Due to its complex structure with several binding moieties, it serves as a promising bioactive agent to be added on surfaces. It has also been previously shown by us that willow and spruce bark extracts that are likely to contain tannins exhibit antibacterial and antiviral efficacy (Pap et al., 2021; Tienaho et al., 2021; Jyske et al., 2023).

Here, we demonstrate that, in addition to being an efficient and safe antiviral in solution for enteroviruses and coronaviruses, TA or tannin containing wood bark and twig extracts can be functionalized to cellulose-based surfaces by using binding polymers to ensure long-term immobilization. For instance, radicalized chitosan was utilized to conjugate tannic acid and tannin-rich extracts effectively to cellulose in order to prevent leaching of the antiviral agents. Excessive leaching could restrict the utilization of the coating especially in hygiene products and food packaging and could also decrease the stability of the antiviral agents (Rakowska et al., 2021; Tarannum and Ahmed, 2023). Chitosan is relatively non-toxic, possesses antimicrobial potential and naturally has attractive interaction with cellulose (Kean and Thanou, 2010; Strnad and Zemljič, 2023). Functionalized surfaces prepared here possessed excellent antiviral efficacy.

As coronaviruses and enteroviruses can persist on cellulose-based materials from hours to several weeks (Abad et al., 1994; Duan et al., 2003; Firquet et al., 2015; van Doremalen et al., 2020), the tannic acid and extract based antiviral coating can remarkably fasten the viral inactivation. Thus, the risk of fomite transmission via contaminated cellulose-based packaging materials could be efficiently reduced without a need for extensive usage of sanitizers that can potentially result in adverse health effects (Saha et al., 2021) and are not designed for cardboard/paper surfaces.

## 2 Materials and methods

### 2.1 Surfaces

Experiments were performed on three different materials: packaging paperboard (Iggesund Incada 175 gsm), cellulose fiber based dry laid material [Sharpcell (SC) 38 gsm], and foam formed sustainable packaging material made of renewable wood fibers [Paptic Tringa (PC) 45 gsm].

### 2.2 Functionalization of surfaces

TA, hydrogen peroxide, ascorbic acid, HMW chitosan, MMW chitosan and citric acid were obtained from Sigma-Aldrich. Industrial polymers used in the binding were styrene maleic anhydride (SMA, Impress SC-745), amphoteric polyvinyl alcohol (C-PVAm, Xelorex RS 1200) and cationic polyacrylamide (C-PAM, Hercobond 2,800-EU). These polymers were utilized to conjugate tannic acid and tannin containing extracts to cellulose. Industrial Norway spruce [*P. abies* (L.) Karst.] bark from a sawmill was retrieved and extracted as described previously (Jyske et al., 2023). Willow (Salix spp.) samples were obtained from Carbons Finland Ltd. Willow bark was obtained by debarking the shoots and willow biomass sample was a combination of bark and woody parts from whole shoot and shoot tips of Klara cultivar. All samples were processed and extracted with a 2 L reactor (Polyclave, Büchi, Switzerland; Raitanen et al., 2020) as described by Tienaho et al. (2021). In short, extraction time was 60 min, temperature 80°C and liquid/solids ratio was 1:10 in all extractions. Extracts were freeze-dried before further experiments.

Compounds were bound to cellulose materials with free radical grafting procedure according to Curcio et al. (2009) using ascorbic acid with hydrogen peroxide. Chitosan was treated with ascorbate radicals to promote covalent bonding with tannic acid and extracts (Table 1). Fibers were impregnated with Cobb-method, with 60 s impregnation time for each fiber type. Samples were dried and heated in an oven (45 s at 140°C) to form covalent bonds. Three different binding approaches were carried out. In the first case, TA and extracts were bound using 5 wt% citric acid and chitosan (MMW) with 0.5:1 ratio (Table 1).

**Table 1:** Experimental set-up binding paperboard with tannic acid, spruce bark extract, and willow extracts.

Sample	Medium	Tannic acid (w/v %)	Spruce bark extract (w/v %)	Willow biomass extract (w/v %)	Willow bark extract (w/v %)	Chitosan (w/v %)	H_2_O_2_-solution (ml)
1	5% (wt) citric acid	0.10				0.05	0.2
2	5% (wt) citric acid	0.50				0.25	1
3	5% (wt) citric acid	1.0				1.25	5
4	5% (wt) citric acid		0.10			0.05	0.2
5	5% (wt) citric acid		0.50			0.25	1
6	5% (wt) citric acid		1.0			1.25	5
7	5% (wt) citric acid			0.10		0.05	0.2
8	5% (wt) citric acid			0.50		0.25	1
9	5% (wt) citric acid			1.0		1.25	5
10	5% (wt) citric acid				0.10	0.05	0.2
11	5% (wt) citric acid				0.50	0.25	1
12	5% (wt) citric acid				1.0	1.25	5

In the second approach (Table 2), fiber samples were coated with tannic acid (0.1–2.5% w/v) using chitosan (LMW) and C-PVAm as binders. Paperboard experiments were conducted with chitosan and C-PVAm (Table 2). Chitosan was used for dry laid material (Sharpcell) and foam formed packaging material (Paptic Tringa). In the third approach, TA concentration was kept 1% (w/v) and, in addition to chitosan, C-PAM and SMA-wax were used as binders (Table 3).

**Table 2:** Experimental set-up binding cellulose materials with tannic acid in combination with C-PVAm or chitosan (CS).

Sample	Medium	Tannic acid (w/v %)	C-PVAm (w/v %)	Chitosan (w/v) %	H_2_O_2_-solution (ml)
13	10% (wt) citric acid	0.1		0.05	0.2
14	10% (wt) citric acid	0.5		0.25	1
15	10% (wt) citric acid	1		0.5	2
16	10% (wt) citric acid	2.5		1.25	5
17	0.0001 M citric acid	0.1	0.25		
18	0.0001 M citric acid	0.5	0.25		
19	0.0001 M citric acid	1.0	0.25		
20	0.0001 M citric acid	2.5	0.25		

**Table 3:** Tannic acid binding experiments using styrene maleic anhydride (SMA), cationized polyacrylamide (C-PAM) and chitosan (CS).

Sample	Medium	Tannic acid (w/v %)	SMA (w/v %)	C-PAM (w/v %)	Chitosan (w/v %)	H_2_O_2_ (ml)	pH
21	Distilled water	1	0.5				7.0
22	5% (wt) citric acid	1	0.5		0.5	2	3.4
23	Distilled water	1		0.1			7.0
24	5% (wt) citric acid	1		0.1	0.5	2	3.4

### 2.3 Cells

Human alveolar basal epithelial adenocarcinoma (A549) cells and MRC-5 cells were obtained from American type culture collection (ATCC). The A549 and MRC-5 cell lines were propagated in Dulbecco’s Modified Eagle Medium (DMEM; Gibco, UK) and Eagle’s Minimum Essential Medium (MEM; Gibco, UK), supplemented with 10% (v/v) Fetal Bovine Serum (FBS, Gibco, UK), 1% (v/v) L-GlutaMAX (Gibco, UK) and 1% antibiotics (v/v; penicillin/streptomycin; Gibco, UK) in an incubator (37°C, 5% CO^2^).

### 2.4 Viruses

Coxsackievirus B3 (CVB3) obtained from ATCC (VR-30) was produced and purified as described before (Myllynen et al., 2016; Ruokolainen et al., 2019), with the only exception of adding 0.1% (v/v) TWEEN® 80 (Sigma-Aldrich, Germany) during the freeze–thaw cycle. For production of seasonal human coronavirus HCoV-OC43 (ATCC, VR-1558), MRC-5 cells were inoculated with HCoV-OC43 at a multiplicity of infection (MOI) of 3 for 2 h at 34°C and replaced with fresh MEM supplemented with 2% FBS and 1% GlutaMAX. Cell culture supernatant was collected 72 h after inoculation. After pelleting of cellular debris, supernatant was stored at −80°C. In order to further purify the crude virus extract for imaging and spectroscopy, a protocol by Dent and Neuman (2015) was used. Briefly, 72 h after the inoculation media was collected and the cell debris was pelleted by centrifugation (JA-10 rotor, 10,000 *g*, 4°C, 20 min). The virus was precipitated using polyethylene glycol 6,000 and NaCl, left on stirring for 30 min at 4°C, and precipitated by centrifugation (JA-10 rotor, 10,000 *g*, 4°C, 30 min). The pellet was dissolved in cold HEPES saline buffer [0.9% NaCl (w/v), 1 mM HEPES, pH 6.7] and concentrated by pelleting through a sucrose gradient (top 10–20 - 30% (w/v) bottom) using a SW-41Ti rotor (100,000 *g*, 4°C, for 120 min). The pellet was dissolved in HEPES saline buffer and stored at −80°C. SARS-CoV-2 (SARS-CoV-2/Finland/1/2020) used was isolated from the first Covid-19 patient in Finland (Haveri et al., 2020).

### 2.5 Virus binding assay and viral infectivity measurements

For determination of antiviral activity, the international standard ISO 18184 was followed with minor modifications. 5-min incubation time of virus was used instead of 2 h (the shortest incubation time suggested in the standard) and the sample size 10 mm x 10 mm differed from 20 mm x 20 mm proposed. Rocking platform was used here to detach viruses as a substitute for vortex mixer. Viral infectivity was determined using the cytopathic effect (CPE) inhibition assay, modified from earlier study (Schmidtke et al., 2001). In the antiviral tests, MRC-5 cells (ATCC, CCL-171; 1.5 × 10^4^ cells/well) or A549 cells (ATCC, CCL-185; 1.2 × 10^4^ cells/well) were seeded into 96-well plates. Cells were then incubated for 24 h in 5% CO_2_ and 37°C. The following day, 10 μl of seasonal human coronavirus HcoV-OC43 (9.0 × 10^5^ PFU/ml or 9.0 × 10^6^ PFU/ml) or CVB3 (2.0 × 10^6^ PFU/ml) was applied on the surface of 1 cm^2^ paperboard pieces for 5 min inside a 12-well plate at room temperature and in humid conditions (RH 90%). 990 μl of culture medium (MEM supplemented with 2% FBS and 1% GlutaMAX or DMEM supplemented with 10% FBS and 1% GlutaMAX) was added and flushed by rocking for 1 min to detach the virus. Collected and diluted media samples were added onto cells. Virus control was prepared by diluting the same amount of virus into cell culturing media that was applied on top of materials. MOIs (0.2 for HCoV-OC43 and 0.1 for CVB3) could be accurately recorded only for virus control samples due to unknown pfu count detached from the materials during flushing. MRC-5 cells were incubated for 5 days at 34°C, while A549 cells were incubated for 48 h at 37°C until a CPE was observed. CPE staining was performed as described (Reshamwala et al., 2021).

### 2.6 Antiviral assay with SARS-CoV-2

Vero-E6 cells (ATCC, CRL-1586) at a density of 5 × 10^4^ cells/well were cultured in a 100 μl of MEM supplemented with 10% FBS, 1% GlutaMAX and 1% penicillin/streptomycin for 24 h at 37°C. Next day, SARS-CoV-2 (SARS-CoV-2/Finland/1/2020; 100 PFU/ml) was pre-treated with samples for 1 h at 34°C and added on cells (MOI 0.0001) for 2 h at 34°C. After adding fresh media, the cells were incubated for 3 days at 34°C. SARS-CoV-2 could have been also amplified at 37°C. Experiments were performed at 34°C based on studies that highlight the effective replication of SARS-COV-2 in epithelial cells in the upper respiratory tract (below 37°C temperature; Wölfel et al., 2020). It was also displayed in a study by Vkovski et al. (2021) that SARS-CoV-2 replicates more efficiently at 33°C compared to 37°C. Then, the supernatant was collected for extraction of viral RNA using a chemagic Viral RNA/DNA kit (PerkinElmer, Turku, Finland). Following the RNA extraction, viral nucleic acid was detected by using SARS-CoV-2 RT-qPCR reagent kit (PerkinElmer, Turku, Finland).

### 2.7 Infection assays for confocal imaging

Ten μl of CVB3 (8.9 × 10^8^ PFU/ml) and HCoV-OC43 (2.6 × 10^7^ PFU/ml) was incubated on surfaces for 5 min and samples were flushed as described before. Collected samples were added onto cells seeded onto a 96-well imaging plate (#655090, Greiner Bio-One) in the previous day. Virus control was prepared by adding the same amount of virus that was used for surfaces. MOIs (6.5 for HCoV-OC43 and 148 for CVB3) could be accurately recorded only for virus control samples due to unknown PFU count detached from the materials. It was ensured that control virus samples were showing decent level of infection for feasible comparison between control and samples with the selected MOI. Infection was allowed to proceed for 5.5 h at 37°C with CVB3 and for 15 h at 34°C with HCoV-OC43 and cells were fixed with 4% (w/v) paraformaldehyde for 30 min.

### 2.8 Immunolabeling and confocal microscopy

The cells were permeabilized with 0.2% Triton-X 100 (w/v) in PBS for 5 min. Cells were treated with primary antibodies diluted in 3% BSA/PBS (w/v) for 1 h. Monoclonal Mouse Anti-Enterovirus Clone 5-D8/1 (Dako Denmark A/S, #M7064) was used for CVB3 and rabbit monoclonal antiserum against the nucleocapsid protein of the HCoV-OC43 (Kolehmainen et al., 2023) was used for HCoV-OC43. Goat anti-mouse 555 (#A21424, Invitrogengen, Thermo Fisher Scientific) and goat anti-rabbit 555 (#A21429, Invitrogen, Thermo Fisher Scientific) were used as secondary antibodies and incubated for 30 min. The DAPI (#D3571, Invitrogen/Molecular Probes) was used to label nuclei. Leica TCS SP8X Falcon microscope (Leica microsystems) was used for imaging. In total 30 images/sample from three experiments were captured corresponding to approximately 650 A549 cells /sample and 1,276 MRC-5 cells/sample in total. CellProfiler 4.2.1. (Stirling et al., 2021) was utilized to count nuclei and infected cells using Otsu thresholding and propagation method, respectively. The infection percentage was calculated by dividing the total number of cells with the number of infected cells. Microscopy images were processed and visualized by using Fiji2.

### 2.9 Quantitative PCR

Quantitative PCR (qPCR) was utilized to determine viral RNA in the flushed samples from the binding experiment. HCoV-43 samples (20 μl) were collected from the flushed media and diluted 1:5 in nuclease-free water (J71768, Thermo Fisher Scientific) and heat-treated for 5 min at 75°C. RNA of CVB3 samples (140 μl) from flushed media was extracted by using QIAamp Viral RNA Mini kit (Qiagen, #52906). Reverse transcription reaction mix included 20 UM-MLV Reverse Transcriptase (#M530A, Promega), RT-buffer (#M530A, Promega), RNase free water (J71768, Thermo Fisher Scientific), 4 U RNAsin ribonuclease inhibitor (#N2515, Promega), 0.5 mM dNTPs (#U1240, Promega), and 1.2 μM HCoV-OC43 reverse primer (5′-AATGTAAAGATGRCCGCGTATT) or enterovirus reverse primer (5′GAAACACGGACACCCAAAGTA) and extracted RNA/heat-treated sample. Reverse transcription was executed at 42°C for 60 min with final 10 min at 70°C. A master mix containing SYBR Green Supermix (#1708886, BioRad), 600 nM HCoV-OC43 forward (5′-TGTTAGGCCRATAATTGAGGAC), and reverse primer (5′-AATGTAAAGATGRCCGCGTATT) or enterovirus forward (5′CGGCCCCTGAATGCGGCTAA) and reverse primer (5′GAAACACGGACACCCAAAGTA) and RNase free water (J71768, Thermo Fisher Scientific) was prepared according to the manufacturer’s instructions. cDNA was combined with reaction mix and it was amplified using qPCR thermal cycler (CFX96TM Real-Time PCR System, Bio-Rad). The qPCR protocol for CVB3 included following amplification steps: 95°C for 10 min; 40 cycles of 95°C for 15 s to 60°C for 1 min, final melt at 72 to 95°C, 1°C/5 s and cooling at 12°C for 10 min. The HCoV-OC43 protocol included following steps: 95°C for 10 min; 40 cycles of 95°C for 15 s to 50°C for 1 min, final melt at 72 to 95°C, 1°C/5 s and cooling at 12°C for 10 min.

C_q_ values acquired from qPCR experiments were used to calculate RNA difference between samples and control virus using following equation that was obtained from a standard curve portraying the duplication of RNA amount each cycle:


$$RNA\;\mathrm{difference}=0.9646e^{0.6948x}\text{,}$$

where x is the difference in C_q_ values between the mean of control virus and samples.

The percentage of viral RNA present in samples was calculated from the equation:


$$\%RNA\;\mathrm{present}=\frac{100}{RNA\;\mathrm{difference}}\text{,}$$

Finally, the percentage of viral RNA bound to tested materials was calculated from the equation:


%RNAbound=100−%RNApresent

### 2.10 Optical density measurements

Different control TA solutions (0.1–1% w/v) were prepared in water. Cellulose materials with bound TA (1 cm^2^) were flushed with 300 μl of H_2_O for 5 min on a rocker inside a 12-well plate. Control and flushed samples were measured for their optical density at 405 nm using Victor X4 2030 Multilabel Reader (PerkinElmer, Turku, Finland).

### 2.11 Contact angle measurements

A water droplet of 40 μl was added on top of cellulose-based reference and functionalized materials. Images of the droplets were taken by using a smartphone camera (Samsung Galaxy A53). The contact angles were measured from the images using GIMP 2.10 software.

### 2.12 Raman spectroscopy

Virus containing samples were prepared by applying 3 μl droplet of purified HCoV-OC43 (6.5 × 10^6^ PFU) on top of paperboard samples (1 cm × 1 cm). Virus was incubated for 5 min on top of samples at RT and 90% relative humidity and then flushed with 1 ml of PBS for 1 min by rocking. PBS was removed and samples were dried in oven at 60°C for 1 h to inactivate remaining viruses. For a positive control sample the same amount of virus was added on gold coated silicon chips. A DXR Raman (Thermo Scientific, USA) with a 50× objective was used for mapping and spectra acquisition. The 785 nm laser with a power of 5 mW was utilized. To acquire spectra of TA, a small amount of powder (#403040, Sigma-Aldrich) was placed on top of microscopy glass.

### 2.13 Statistical analysis

A statistical analysis of the CPE and confocal microscopy experiments was performed using GraphPad Prism 6 (GraphPad Software, San Diego, CA, USA). The statistical significance was calculated by performing one-way ANOVA, followed by the Bonferroni test. The 50% effective concentrations (EC_50_) and 50% cytotoxic concentrations (CC_50_) were calculated by non-linear regression analysis of the dose–response curves.

## 3 Results

### 3.1 Tannic acid and spruce bark extract show efficacy against non-enveloped and enveloped viruses

**Figure 1:**
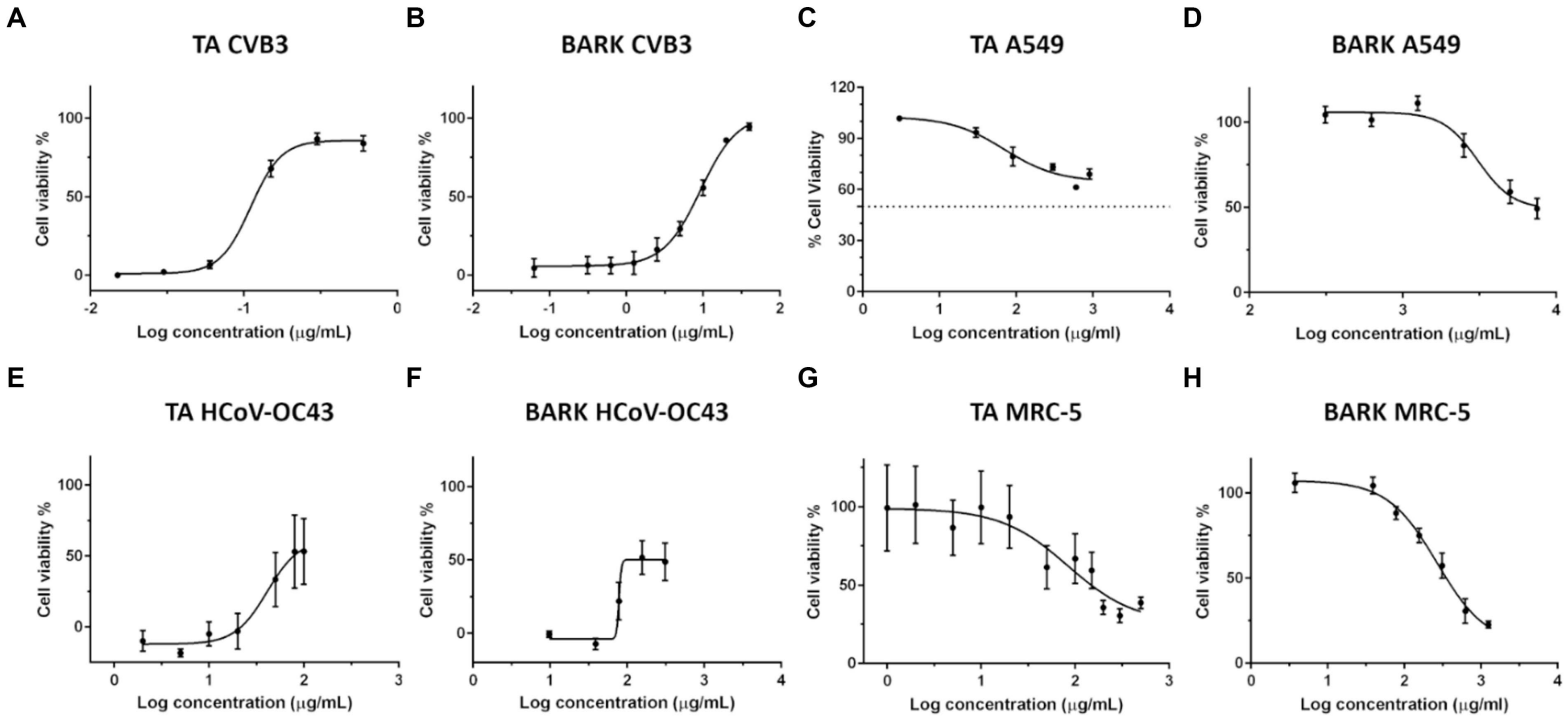
Dose–response curves for determination of 50% effective concentration (EC_50_) of tannic acid (TA) and spruce bark extract against viruses and 50% cytotoxic concentration (CC_50_). Antiviral efficacy of TA **(A,E)** and spruce bark extract **(B,F)** was determined against CVB3 **(A,B)** and HCoV-OC43 **(E,F)**. CVB3 titre in the virus-compound mix was 2 × 10^7^ PFU/ml, while the MOI was 10. HCoV-OC43 titre in the virus-sample mix was 1.6 × 10^3^ PFU/ml and final MOI was 0.008. Toxicity of TA **(C,G)** and spruce bark extract **(D,H)** was studied on A549 and MRC-5 cells, respectively. All the experiments were carried out using the CPE inhibition assay. Concentrations of TA and spruce bark extract are represented as Log (10) of μg/ml on the *x*-axis. The results are expressed as average values ± standard error of the mean (SEM).

The first aim was to evaluate the antiviral potential of the commercially available TA and the produced spruce bark extract for both enveloped and non-enveloped viruses. CVB3 was schosen as an example of non-enveloped enteroviruses and the seasonal HCoV-OC43 as an example of human beta coronaviruses. The screening of TA and spruce bark extract against enterovirus (Figures 1A,B) and seasonal coronavirus (Figures 1E,F) showed that both the compound mixtures were effective in protecting the cells from enveloped as well as non-enveloped viruses.

To further demonstrate the antiviral potency of both these preparations, their 50% effective concentration (EC_50_) was determined by performing non-linear regression analysis from their dose–response curves (Table 4). Based on EC_50_ values, the efficacy of TA was superior by 70-fold compared to spruce bark extract against CVB3. In case of seasonal coronavirus, the efficacy of both the compound mixtures were in the similar range. Overall, both the samples were more effective against the enterovirus as compared to the enveloped coronavirus. Cytotoxicity of the samples was also studied (Figure 1) and 50% cytotoxic concentrations (CC_50_) were calculated for TA and spruce bark extracts (Table 4). Both the preparations showed toxicity to MRC-5 cells at higher concentrations. Comparatively, those higher concentrations were well tolerated by A549 cells. Selectivity Index (SI) calculated from ratio of CC_50_ and EC_50_ demonstrate the strong antiviral potential of both these samples.

**Table 4:** Antiviral activity and cytotoxicity of TA and spruce bark extract.

Virus	Sample	EC_50_ (μg/ml)	CC_50_ (μg/ml)	SI
CVB3	TA	0.12	Incalculable	high
HCoV-OC43	TA	78.16	148.25	1.89
CVB3	Spruce bark extract	8.41	8892.01	1057.31
HCoV-OC43	Spruce bark extract	95.49	353.18	3.69

The preparations were also tested against the virulent SARS-CoV-2. Cq values determined from qPCR gives a measure of viral replication in the cells. As Cq values are inversely proportional to the amount of viral RNA (cDNA) present in the sample, the lower the Cq values, the higher the amount of RNA present and vice versa. The virus control had a Cq value of 14.82, indicating a high amount of viral RNA. However, the Cq values for the virus pre-incubated with 100 μg/ml of either of the samples was significantly higher implying a significant reduction in the viral RNA (Table 5). Cq values were also used to calculate the logarithmic reduction of the viral infectivity (Table 5). Based on these calculations, an outstanding 5.8-to 7-log decline was observed in the viral RNA when virus was pre-incubated with the compound mixtures. In conclusion, both TA and spruce bark extract demonstrated excellent antiviral efficacy against SARS-CoV-2.

**Table 5:** The effect of TA and spruce bark extract (100 μg/ml) pre-treatment on SARS-CoV-2.

Sample	Cq mean value	Difference in Cq value compared to VC	RNA difference	Log difference
TA	33.95	19.13	571589.88	5.75
Spruce bark extract	37.90	23.08	8913422.35	6.95
VC	14.82	-	-	-

Vero E6 cells were infected with pre-treated SARS-CoV-2 virus. Cq mean values of the test and virus control (VC) samples obtained from the qPCR are shown. These mean Cq values were used to calculate the difference between test and virus control samples, which was further used to calculate logarithmic RNA difference.

### 3.2 Viruses remain infectious on reference samples during 5-min incubation

**Figure 2:**
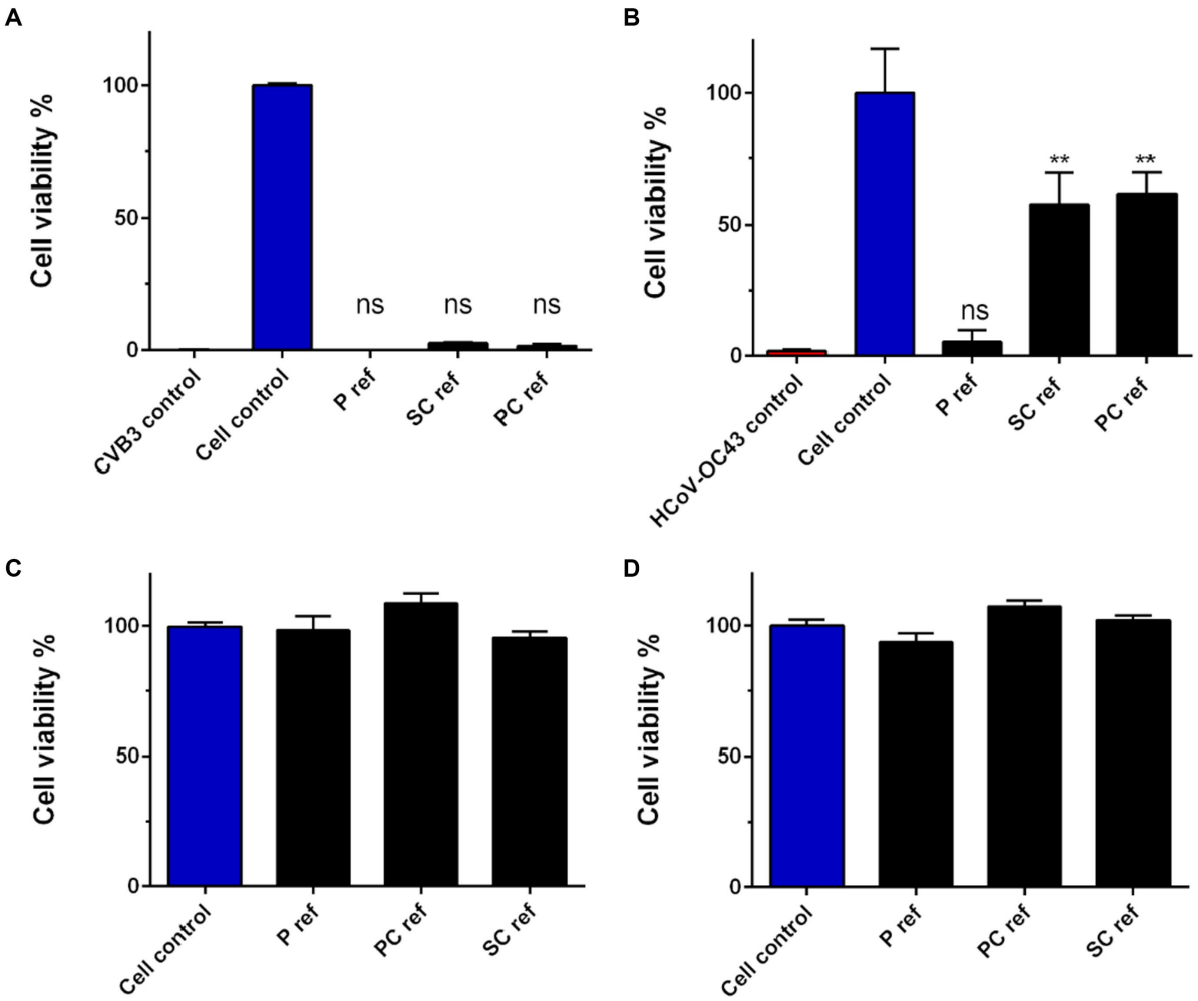
Infectivity of HCoV-OC43 and CVB3 on cellulose-based reference materials **(A,B)** after 5-min incubation at RT and toxicity of materials on cells **(C,D)**. **(A)** Ten μl of CVB3 (2.0 × 10^6^ PFU/ml) and **(B)** HCoV-OC43 (9.0 × 10^6^ PFU/ml) were applied on cellulose-based paperboard (P), Sharpcell (SC) and Paptic (P) reference materials (ref) without any additional treatment and CPE assay was exploited to determine the viral infectivity due to treatment. Sample treatments and virus control are normalized against cell control without any infection. Results are presented as average values of 3 biological and 3 technical replicates of each sample ± standard error of the mean (SEM). * *p* < 0.05, ** *p* < 0.01, *** *p* < 0.001 and **** *p* < 0.0001 versus the virus control (analyzed with one-way ANOVA with Bonferroni test). Toxicity of reference materials was studied on A549 **(C)** and MRC-5 **(D)** cells using CPE assay.

To begin with, it was explored whether the cellulose-based reference materials (paperboard, SC or PC) possessed any antiviral properties without additional treatments. To test the antiviral activity, harsh conditions were used, only 5-min incubation at room temperature. The results indicated that none of the untreated reference materials showed antiviral activity against the stable non-enveloped CVB3 during 5-min incubation (Figure 2A). Also, the enveloped seasonal coronavirus, HCoV-OC43, remained infectious on paperboard in tested conditions (Figure 2B). PC and SC materials showed moderate antiviral effect against the coronavirus (Figure 2B). As the infectivity assay employed is cell-based, anything toxic that could dissolve from the samples could contribute to the results. Thus, all the samples and test conditions were studied here and none of them were toxic to MRC-5 nor A549 cells (Figures 2C,D).

### 3.3 Tannic acid treated paperboard reduces infectivity of CVB3 and HCoV-OC43

**Figure 3:**
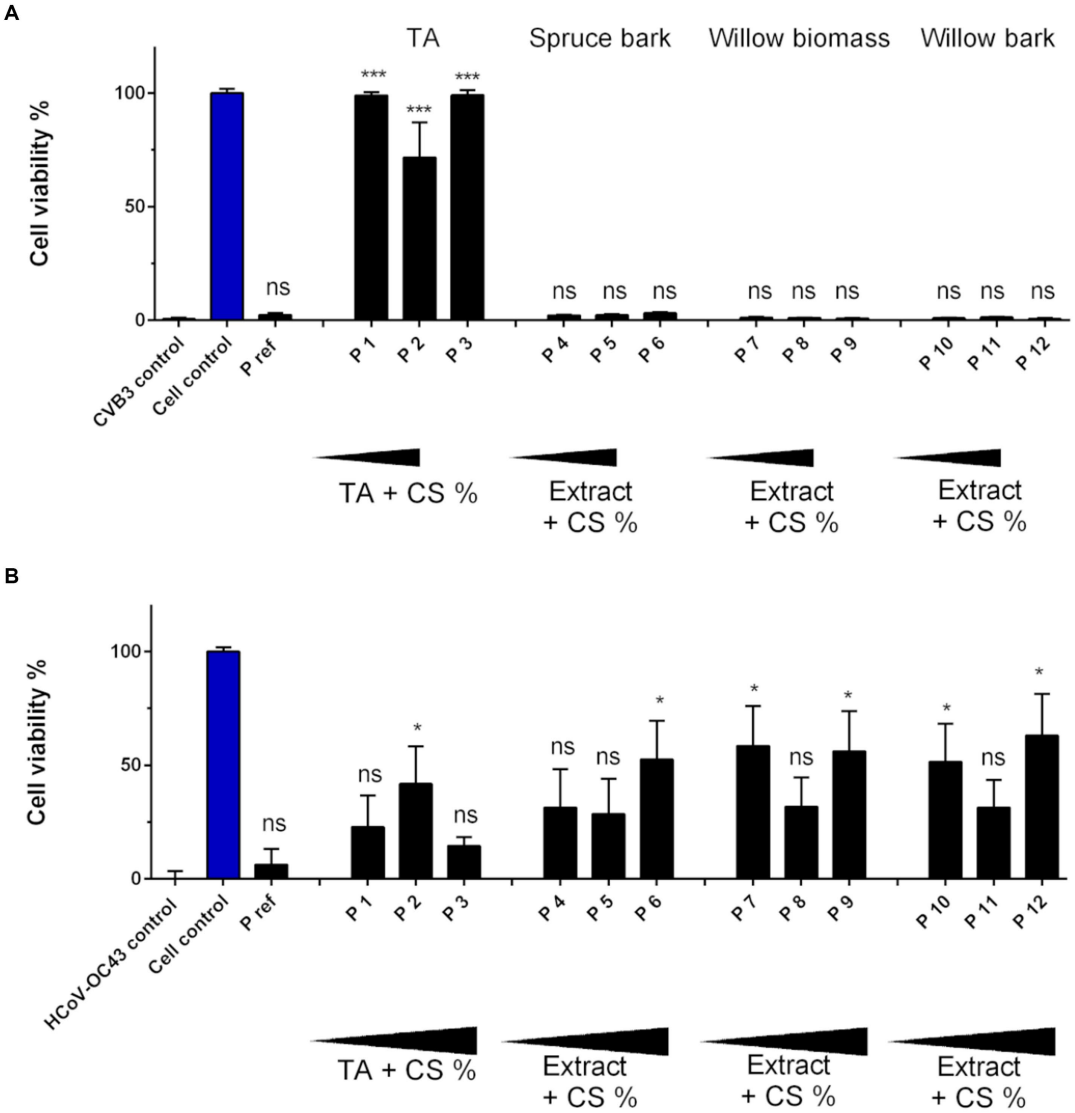
Infectivity of CVB3 and HCoV-OC43 after 5-min incubation on paperboard functionalized with tannic acid (TA) and wood extracts. **(A)** Ten μl of CVB3 (2.0 × 10^6^ PFU/ml) and **(B)** HCoV-OC43 (9.0 × 10^6^ PFU/ml) were applied on paperboard materials for 5 min at RT functionalized with TA (P 1–3), spruce bark extract (P 4–6), willow biomass extract (P 7–9) and willow bark extract (P 10–12) in combination with chitosan (CS). Viral infectivity was studied using CPE assay. Increasing amounts of functionalization materials were tested. Detailed chemical composition of each solution used in functionalization is presented in Table 1. Sample treatments and virus control are normalized against cell control without any virus infection. Results are presented as average values of 3 biological and 3 technical replicates of each sample ± standard error of the mean (SEM). * *p* < 0.05, ** *p* < 0.01, *** *p* < 0.001 and **** *p* < 0.0001 versus the virus control (analyzed with one-way ANOVA with Bonferroni test).

To functionalize the cellulose material TA was first tested in combination with chitosan (CS) against viruses. TA was bound to paperboard in concentrations ranging from 0.1 to 1% (w/v) with increasing concentrations of CS (0.05–1.25% w/v; Detailed composition of the samples is shown in Table 1). CS and other binding polymers were utilized to achieve stable binding of TA into cellulose. CVB3 was added again on the material for just 5 min at RT to see the efficacy after only a short encounter with the material. The results with CVB3 demonstrated remarkably good efficacy with all used concentrations of TA (Figure 3A). In contrast, in the similar setting, willow and spruce extracts showed no antiviral effect against CVB3 on paperboard during 5-min incubation suggesting that at least the tested concentrations had no efficacy against CVB3 when bound to paperboard (Figure 3A). Interestingly, the outcome with HCoV-OC43 was quite different: TA showed moderate but varying activity against coronaviruses on paperboard, and there was no major difference between TA and extract containing samples (Figure 3B). There was also no clear dose–response between TA/extract and CS concentrations for HCoV-OC43.

### 3.4 Chitosan and C-PVAm as binding polymers

**Figure 4:**
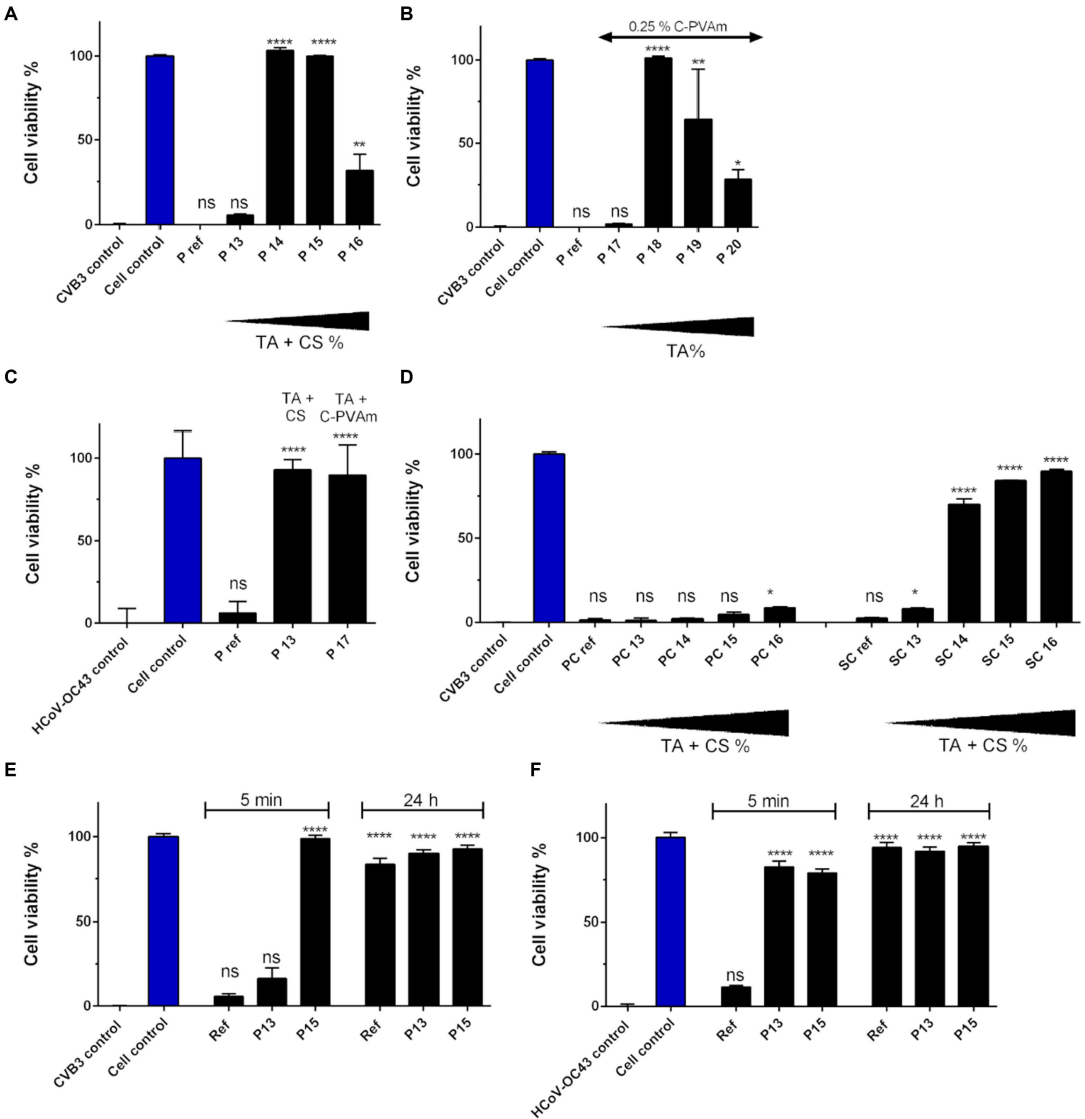
Infectivity of HCoV-OC43 and CVB3 after a 5-min incubation on cellulose-based materials functionalized with tannic acid (TA) and chitosan (CS) or C-PVAm. **(A)** Ten μl of CVB3 (2.0 × 10^6^ PFU/ml) was incubated for 5 min at RT on reference paperboard (P) and paperboard functionalized with different concentrations of TA and CS (P 13–16) or **(B)** C-PVAm (P 17–20). **(C)** Viral infectivity of HCoV-OC43 (9.0 × 10^5^ PFU/ml) was studied on paperboard functionalized with 0.1% TA solution in combination with CS (P 13) and C-PVAm (P 17). **(D)** Viral infectivity of CVB3 determined after incubation on Paptic (PC) and Sharpcell (SC) materials functionalized with TA and CS. Viral infectivity of **(E)** CVB3 (2.0 × 10^6^ PFU/ml) and **(F)** HCoV-OC43 (9.0 × 10^5^ PFU/ml) was determined after 24 h incubation on reference, P13 (0.1% TA) and P15 (1%) paperboard. Efficacy of 5-min treatment was also confirmed at the same time for accurate comparison. Detailed chemical composition of each solution used in functionalization is presented in Table 2. Sample treatments and virus control are normalized against cell control without any virus infection. Results are presented as average values of 3 biological and 3 technical replicates of each sample ± standard error of the mean (SEM). * *p* < 0.05, ** *p* < 0.01, *** *p* < 0.001 and **** *p* < 0.0001 versus the virus control (analyzed with one-way ANOVA with Bonferroni test).

**Figure 5:**
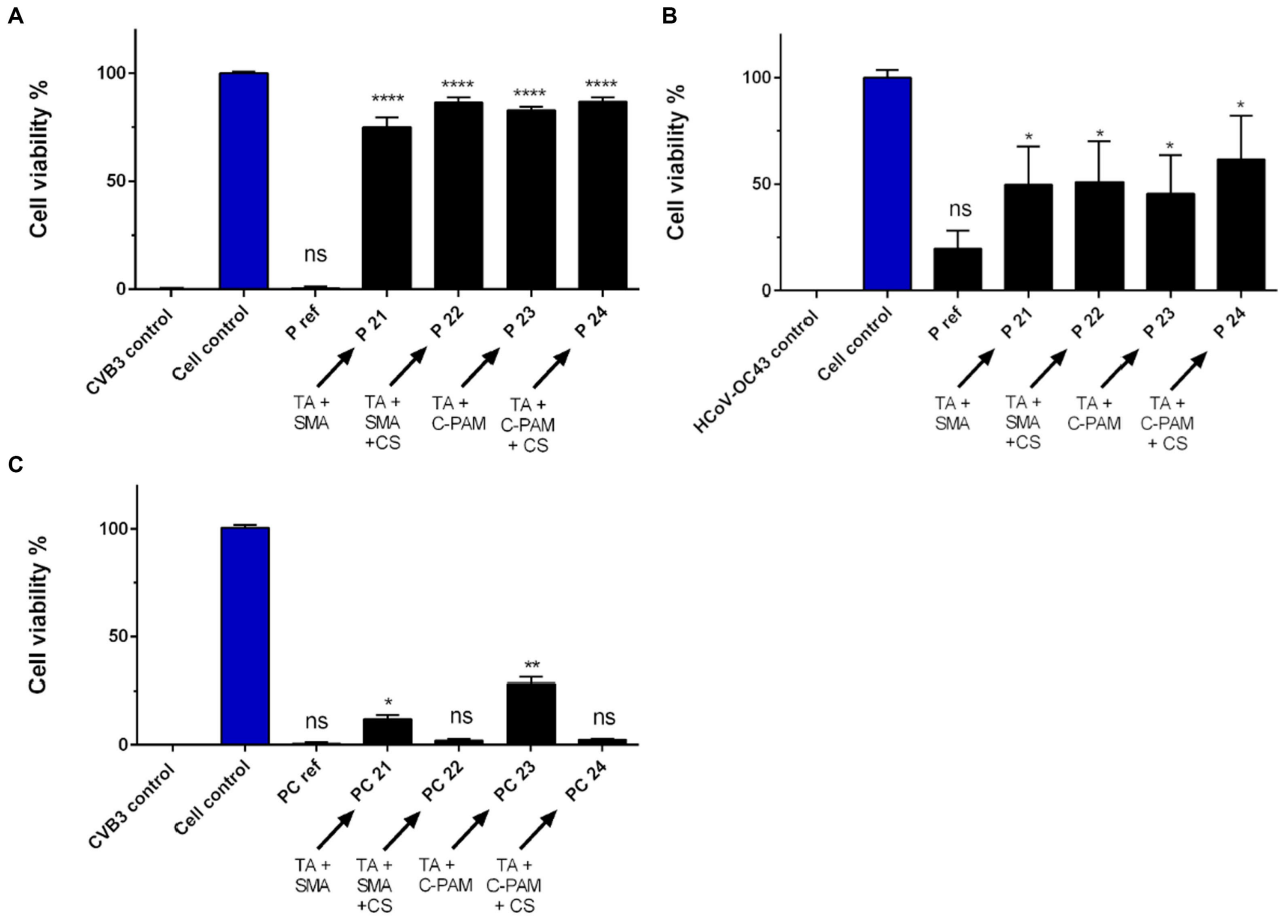
SMA and C-PAM are tested as binding partners for antiviral functionalization. **(A)** Ten μl of CVB3 (2.0 × 10^6^ PFU/ml) and **(B)** HCoV-OC43 (9.0 × 10^5^ PFU/ml) were applied for 5 min at RT on reference paperboard (P) and paperboard functionalized with 1% tannic acid (TA), SMA or C-PAM and with or without chitosan (CS). **(C)** Viral infectivity of CVB was studied after 5-min incubation on Paptic (PC) material functionalized with 1% TA solution, SMA or C-PAM, and with or without CS. Detailed chemical composition of each solution used in functionalization is presented in Table 3. Sample treatments and virus control are normalized against cell control without any virus infection. Results are presented as average values of 3 biological and 3 technical replicates of each sample ± standard error of the mean (SEM). * *p* < 0.05, ** *p* < 0.01, *** *p* < 0.001 and **** *p* < 0.0001 versus the virus control (analyzed with one-way ANOVA with Bonferroni test).

Paperboard showed significant antiviral activity against CVB3 when it had been impregnated with 0.5% (P 14 in Figure 4A) and 1% tannic (P 15) acid (w/v) in combination with 1:2 CS (Figure 4A; Detailed sample composition is shown in Table 2). Surprisingly, increasing TA percentage to 2.5% (w/v) and CS up to 1.25% (w/v) led (P 16) to a decrease in antiviral activity, suggesting that the high concentrations of CS, TA and hydrogen peroxide combined were not anymore optimal for gaining antiviral functionality. A different binding partner for TA was tried next, namely C-PVAm instead of CS (Figure 4B). The greatest antiviral effect on paperboard was achieved by using 0.5% (w/v) TA solution (P 18) with 0.25% (w/v) C-PVAm for preparation. Like with CS, high amount of TA (2.5%) with C-PVAm resulted in no antiviral efficacy against CVB3 (P 20 in Figure 4B). However, results indicated that when combined with CS there was a wider range of efficient TA concentrations against CVB3 compared to combination of C-PVAm and TA. Also, this time already 0.1% TA containing paperboard (P 13 and P 17 in Figure 4C) was able to totally inhibit HCoV-OC43 infectivity when viral amount on top of the sample was diluted 1:10 compared to the previous test shown in Figure 3B. In the case of HCoV-OC43, there was no significant difference whether CS or C-PVAm was used in combination with TA. Functionalized PC material did not provide any notable antiviral effect against CVB3 (Figure 4D). Instead, remarkable antiviral action against CVB3 with increasing dose–response was detected following treatment on SC material having 0.5–2.5% TA content (SC 14–16 in Figure 4D). These results demonstrate that the methods used for TA binding do not work on all cellulose-based materials but rather efficacy is affected by the differences in the composition of the cellulose-based material itself. Later the viral infectivity was determined on reference, P13 and P15 paperboard after 24-h incubation. The infectivity was also investigated again after 5-min exposure to secure proper comparison. The results of 5-min treatment were similar with previous findings. CVB3 infectivity was lost on 1% TA paperboard (P15), but the virus remained infectious on the reference material (Figure 4E). HCoV-OC43 infectivity was inhibited already with 0.1% TA paperboard (P13), whereas the virus was still infectious on the reference material (Figure 4F). The results of 24-h incubation displayed that infectivity of both viruses was inhibited on TA treated paperboards (P13 and P15), but also on the reference material (Figures 5E,F).

### 3.5 SMA and C-PAM are equally good binding polymers for TA driven antiviral functionalization

Next, the antiviral effect of (1%) TA functionalization on paperboard in combination with SMA and C-PAM (+/− CS) was studied against CVB3 (Figure 5A; Detailed sample composition is shown in Table 3). All the treatments provided strong antiviral effect against CVB3. There was no statistically significant difference in the antiviral effect between the used binding polymers. Neither pH of stock solution nor involvement of CS caused any difference. When the similarly treated paperboard samples were tested against HCoV-OC43, the incubation on functionalized paperboard led to moderately increased antiviral effect, but there was no significant difference between the treatments (Figure 5B).

When PC material was treated with 1% TA treatment in combination with these different binding polymers, there was no remarkable antiviral effect against CVB3 with most of the samples (Figure 5C). However, there was a small increase in the efficacy observed when either SMA or C-PAM were used without CS involved.

### 3.6 Confocal microscopy study on virus infectivity

**Figure 6:**
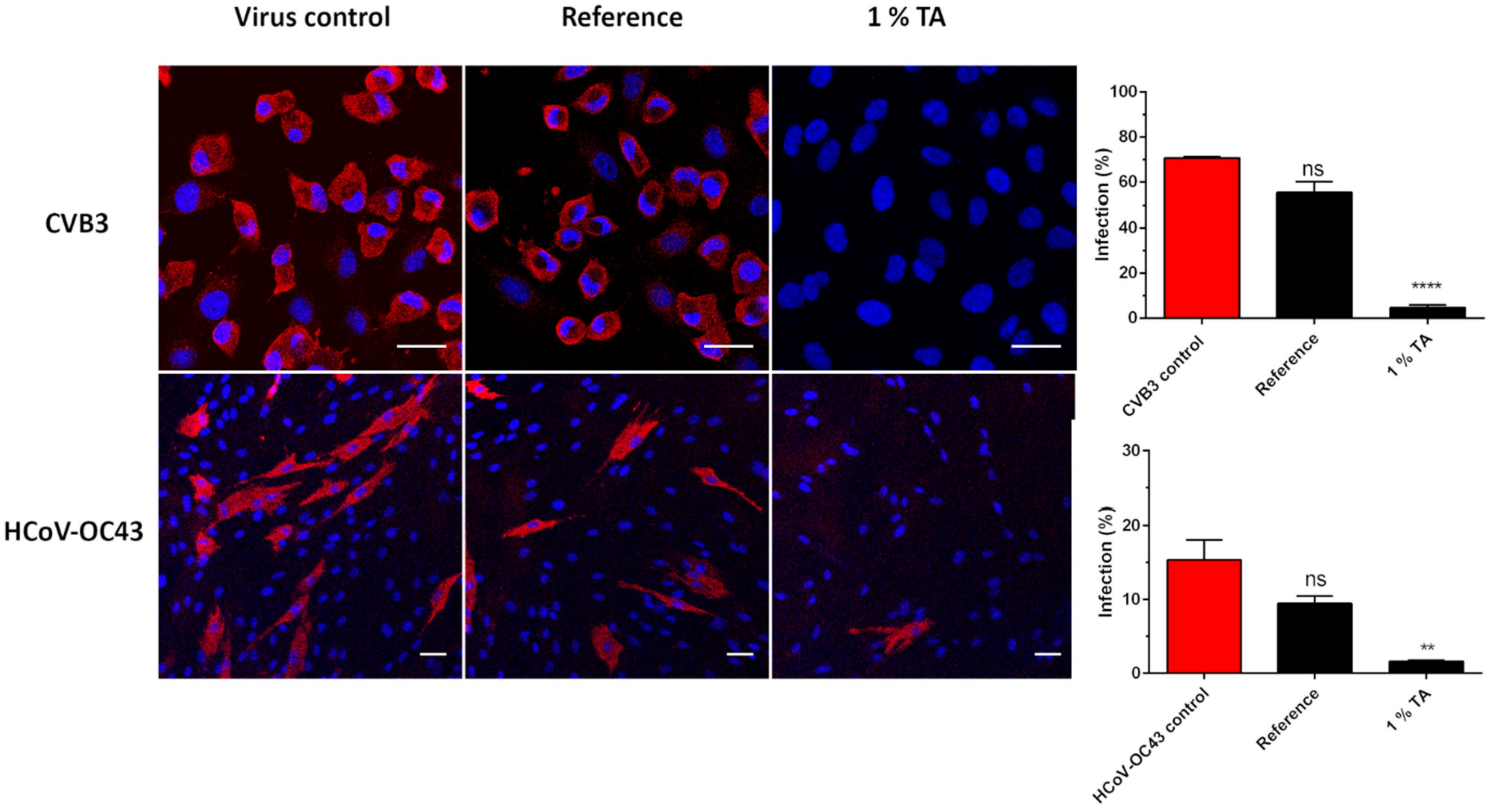
Confocal microscopy study of virus treatment on functionalized paperboard for 5 min at RT. After a 5-min treatment of ten μl of CVB3 (8.9 × 10^8^ PFU/ml) and HCoV-OC43 (2.6 × 10^7^ PFU/ml) on three replicates of 1% TA functionalized (P15 in Table 2) and reference paperboard viruses were flushed from the surfaces and applied on cells to determine their infectivity. CVB3 infected A549 cells were labelled for VP1 capsid protein (red) after 5.5 h of infection, while HCoV-OC43 infected MRC-5 cells were labelled for nucleocapsid protein (red) after 15.5 h of infection. DAPI stained cell nuclei are in blue. Scale bars, 30 μm (top) and 50 μm (down). Quantification of the imaging data with CellProfiler is calculated of approximately 650 and 1,276 cells for CVB3 and HCoV-OC43 samples, respectively. * *p* < 0.05, ** *p* < 0.01, *** *p* < 0.001 and **** *p* < 0.0001 versus the virus control (analyzed with one-way ANOVA with Bonferroni test).

Confocal immunofluorescence microscopy was employed to confirm the state of viral infectivity following treatment on reference and 1% TA functionalized paperboard (P 15). A droplet of CVB3 was incubated on top of samples for 5 min at RT at 90% RH and then the virus was flushed with cell culturing media. Flushed media was added to A549 cells and after 5.5-h infection at 37°C the cells were fixed and permeabilized. Next, the cell nuclei and VP1 capsid protein of CVB3 were immunolabeled to visualize infected cells that produced high amounts of new capsid proteins. By confocal imaging it was possible to directly monitor what cells were infected. When observed visually there was no significant difference in the amount of CVB3 infected A549 cells between virus control and virus treated on top of reference paperboard (Figure 6). Instead, after virus treatment on TA functionalized sample, few infected cells were observed (Figure 6). Quantification of the imaging data revealed that 70.6% of the cells treated with control virus were infected and after 5-min treatment of CVB3 on reference paperboard, the virus infectivity was still high as 55.4% of the cells were infected (Figure 6). In contrast, only a 5-min incubation on 1% TA paperboard caused a strong reduction in infectivity as only 4.9% of the cells were infected due to the treatment (Figure 6). Thus, viral infectivity was reduced 93.1% compared to the control virus, indicating that the viral protein translation was efficiently blocked during one virus life cycle (5.5 h).

Next, the capability of HCoV-OC43 to infect MRC-5 cells after short incubation on 1% TA functionalized and reference paperboard samples was studied next. As the infection cycle is typically longer than for enteroviruses, the infection was studied 15 h post infection (p.i.) For HCoV-OC43, viral nucleocapsid protein was visualized by immunolabeling to see if viruses had produced high amount of new protein as a proof of active replication and translation. There was no apparent difference in the infectivity between cells infected with the control virus and the virus incubated on reference sample as was expected based on the CPE tests (Figure 6). On the contrary, virus treatment on TA functionalized paperboard had mostly prevented HCoV-OC43 infection in MRC-5 cells (Figure 6). Quantification of the results showed that 15.5 and 9.5% of cells were infected with the untreated control virus and the virus treated on reference sample for 5 min, respectively (Figure 6). In contrast, only 1.6% of cells were infected after 5-min treatment of virus on 1% TA paperboard (Figure 6). Thus, the viral infectivity was reduced 89.6% compared to control virus, indicating that the viral protein translation was efficiently blocked during one virus life cycle (15 h). Altogether, confocal microscopy confirmed the results gained from CPE staining: infectivity of HCoV-OC43 and especially CVB3 was heavily reduced after 5-min incubation on 1% TA paperboard (P 15).

### 3.7 Enteroviruses bind to TA functionalized surfaces more strongly than coronaviruses

**Figure 7:**
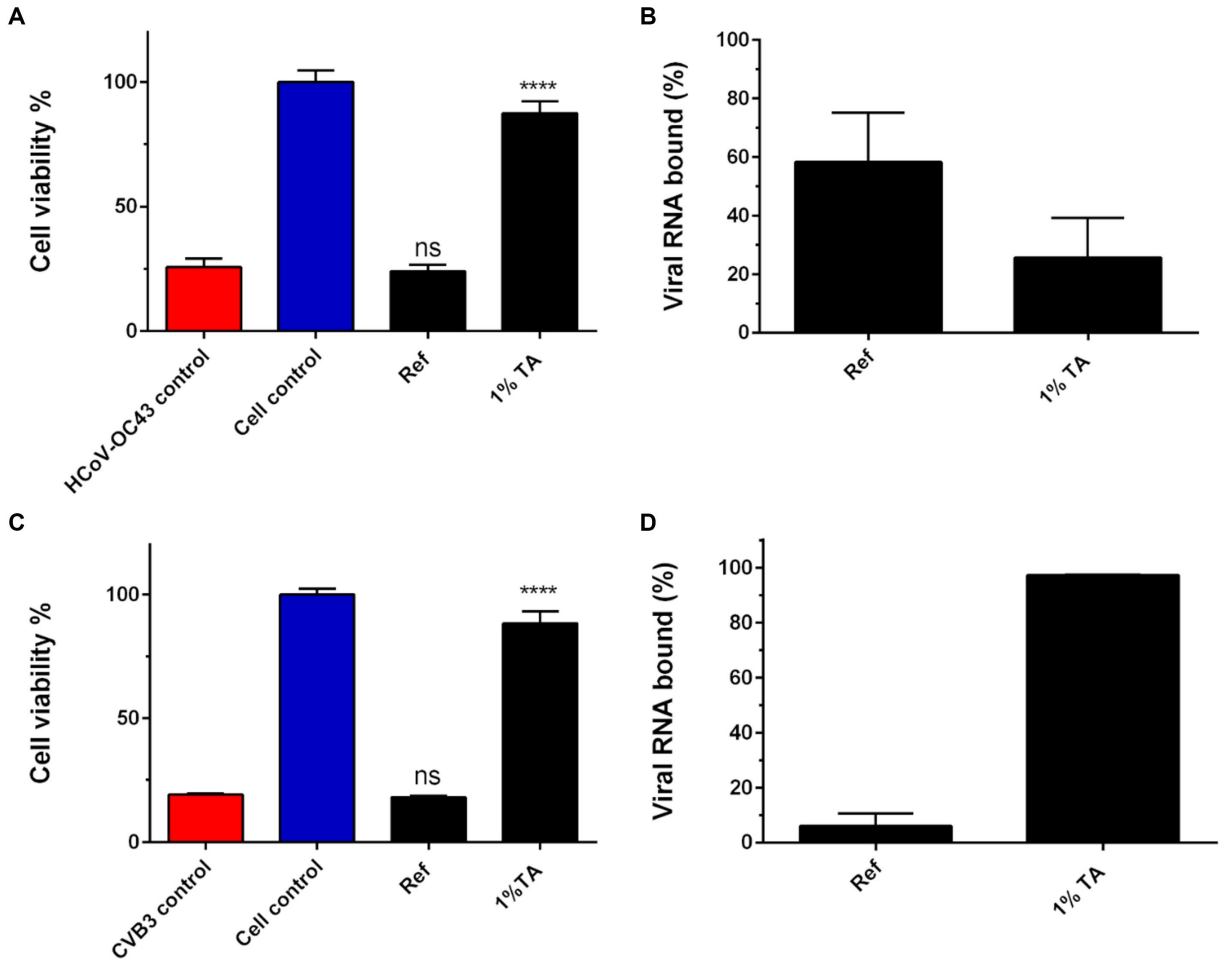
A study on an ability of functionalized material to bind and inactivate viruses. Ten μl of HCoV-OC43 (9.0 × 10^5^ PFU/ml; **A,B**) and CVB3 (2.0 × 10^6^ PFU/ml; **C,D**) were applied on reference (Ref) or TA functionalized paperboard (P 15 in Table 2) for 5 min. Viruses were flushed from the surfaces and both the infectivity **(A,C)** and the amount of viral RNA **(B,D)** were evaluated of the flushed viruses with CPE and qPCR assays, respectively. qPCR results are presented as a percentage of viral RNA bound to the paperboard materials. Sample treatments and virus control are normalized against cell control without any virus infection. Results are presented as average values of 3 biological and 3 technical replicates of each sample ± standard error of the mean (SEM). * *p* < 0.05, ** *p* < 0.01, *** *p* < 0.001 and **** *p* < 0.0001 versus the virus control (analyzed with one-way ANOVA with Bonferroni test).

After displaying the antiviral efficacy of TA containing paperboard it was unclear whether the viruses or viral RNA was tightly bound to functionalized paperboard due to 5-min incubation on sample or if the virus/viral RNA could be easily flushed from the material. Thus, viruses were again applied on the functionalized and reference paperboard for 5-min treatment followed by flushing the virus from the surface. qPCR was then conducted to determine the amount of viral RNA in flushed media and subsequently the percentage of viral RNA that was still bound to the materials. As a positive control there was untreated virus with the same PFU count that was originally placed on the materials. Also, viral infectivity was determined with CPE assay from the same flushed media as we wanted to verify the state of the viral infectivity from the same experiment to rule out any erroneous conclusions. Results of infectivity measurements established that TA functionalized paperboard (P 15) possessed strong antiviral efficacy against HCoV-OC43 (Figure 7A). Consistent with the earlier experiments, HCoV-OC43 was still fully infectious on reference paperboard after a short incubation period. Interestingly, the results of qPCR quantification implied that 58.4% of viral RNA of HCoV-OC43 (58.4%) was bound to the reference paperboard following the 5-min treatment and flushing, whereas less viral RNA was bound to the functionalized paperboard (25.7%) (Figure 7B).

Infectivity measurements also confirmed the potent antiviral efficacy of TA functionalized paperboard against CVB3 after 5-min incubation (Figure 7C). In contrast to the results with HCoV-OC43, the qPCR quantification indicated that high amount of the viral RNA of CVB3 (97.2%) was bound to the functionalized paperboard (Figure 7D). This implies that most of the viral particles were bound to the functionalized material following the 5-min incubation and flushing. On the other hand, only minor amount (6.2%) of the viral RNA of CVB3 was bound to the reference paperboard, while the virus was also fully infectious after 5-min treatment on it.

**Figure 8:**
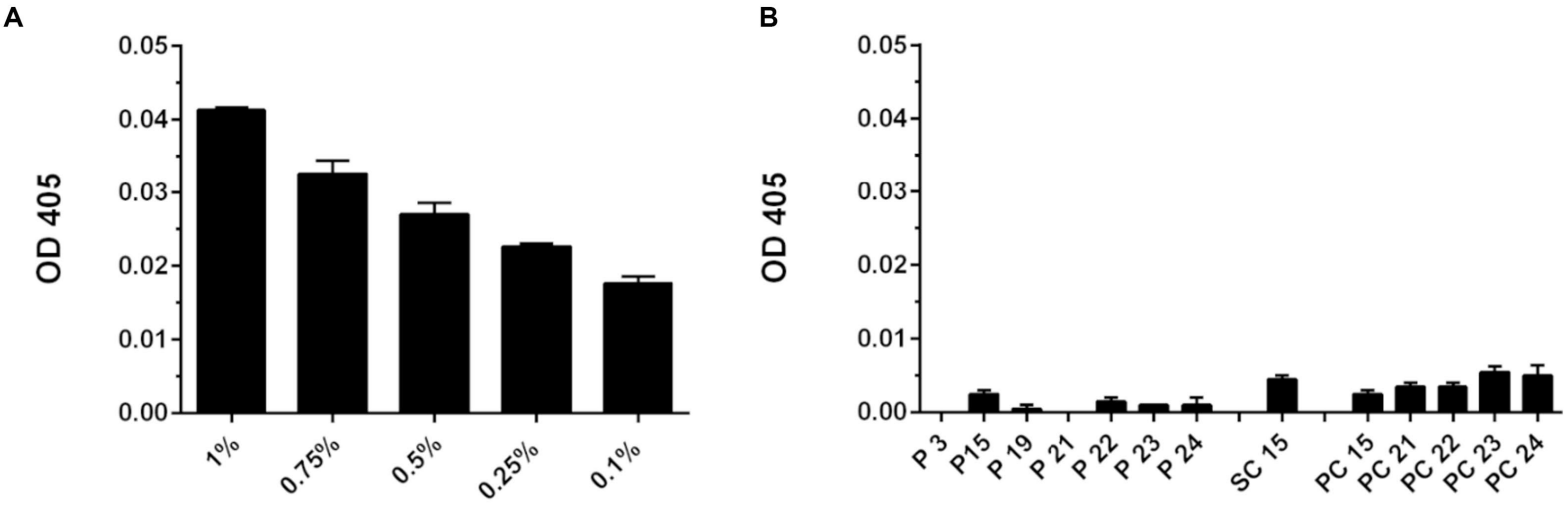
Optical density at 450 nm (OD 405) determined for tannic acid and flushed samples. **(A)** Different concentrations of TA solution were measured to determine OD 405 that correlates with the amount of TA present. **(B)** Cellulose materials functionalized with 1% TA were flushed with H_2_O for 5 min and OD 405 was measured to resolve whether any TA was present in liquid after flushing. Blank value of H_2_O was subtracted of each OD 405 value measured. Results are presented as average values of 3 replicates of each sample ± standard error of the mean (SEM).

Next, it was studied if there was any leakage of TA from the materials treated with 1% TA that could inhibit viruses in soluble form already in the near vicinity of the cellulose material. Thus, optical density at 405 nm of different tannic acid concentrations was measured first for comparison (Figure 8A). As 1% TA was used for functionalization with these samples, it was also kept as the maximum amount of TA that could be leaked from the materials during 5-min incubation. Next the 1% TA containing materials were flushed with H_2_O for 5 min and optical density at 405 nm of the liquids was measured. The results indicated that only a minor amount of TA was present in liquids after flushes and there was no significant difference in the amount of released TA between different materials and treatments (Figure 8B).

To understand whether cellulose-based materials were hydrophobic or hydrophilic, a contact angle of water droplet was measured on 3 reference and 1% TA treated materials post 5-min incubation. It was observed that reference SC material absorbed water droplet immediately being the most hydrophilic of all 3 materials (data not shown). On the other hand, the contact angle of water droplet was 96° on refence paperboard, whereas PC material showed the highest level of hydrophobicity as water droplet had 107° contact angle on the material. After functionalization with 1% TA the SC material (SC 15) still absorbed all the water instantly while contact angle of water droplet on PC material (PC 15) was 102° indicating a high level of hydrophobicity. Interestingly, the droplet contact angle on functionalized paperboard (P 15) was now 56° suggesting that TA functionalization increased its hydrophilicity.

### 3.8 Raman spectroscopy shows colocalization of HCoV-OC43 and TA on a functionalized paperboard

**Figure 9:**
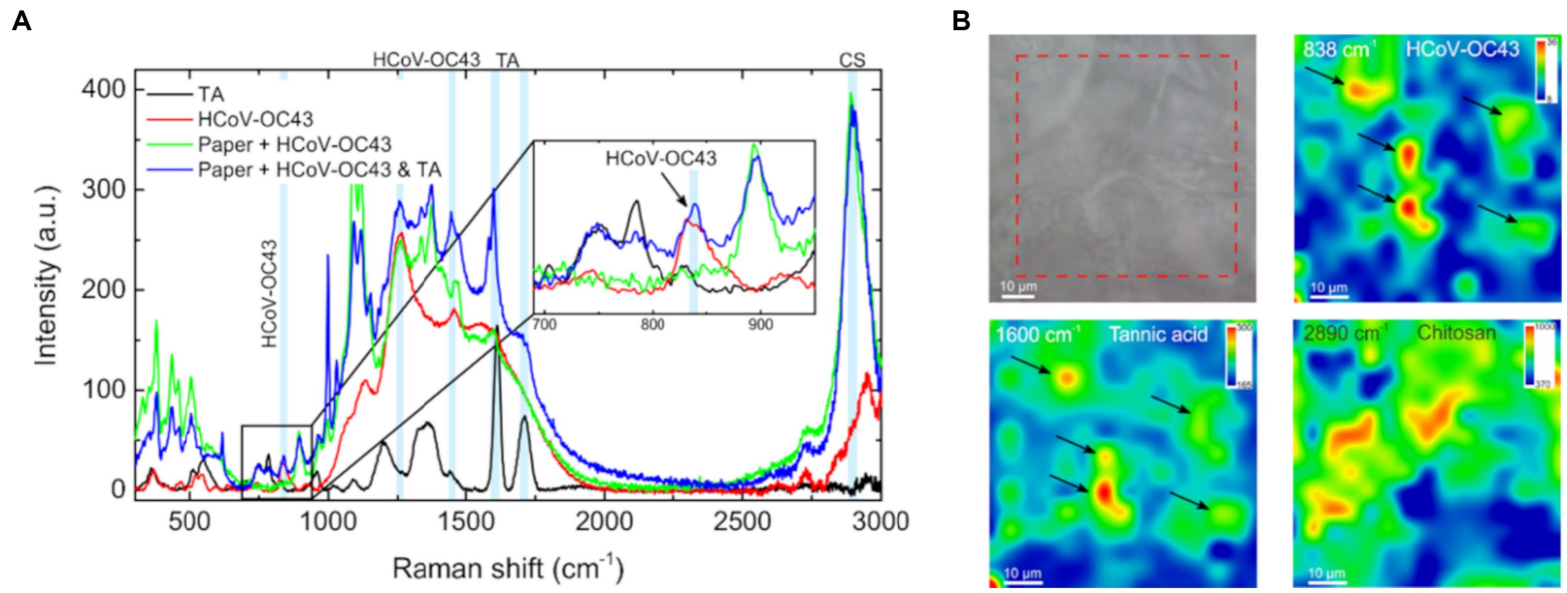
Raman mapping of tannic acid, chitosan and HCoV-OC43 on functionalized material. **(A)** Raman signature spectra for TA powder and purified HCoV-OC43 on gold surface, as well as for HCoV-OC43 on paperboard with (P 15 in Table 2) and without TA. Three signature peaks for HCoV-OC43 (838, 1,250, and 1,450 cm^−1^), two signature peaks for TA (1,600 and 1710 cm^−1^) and one for CS (2,890 cm^−1^) are indicated in the spectra. **(B)** Raman mapping of the TA functionalized paperboard with HCoV-OC43 bound for 5 min. An optical image visualizes the area mapped. Mapping of signature peaks of HCoV-OC43 (838 cm^−1^), TA (1,600 cm^−1^) and chitosan (2,890 cm^−1^) from the same area.

We wanted to characterize the TA binding to the paperboard material further and used Raman spectroscopy for that. First, the Raman spectra were measured for the TA powder and inactivated HCoV-OC43 separately to define the most intense Raman bands for their specific detection on a paperboard (Figure 9A). For TA, two fingerprints in the Raman spectrum as 1,600 cm^−1^ (C=C bond) and 1,710 cm^−1^ (C=O bond; Huguenin et al., 2015) were chosen. Several prominent bands were found for HCoV-OC43 on gold surface such as 838 cm^−1^ (tyrosine, valine, isoleucine), 1,250 cm^−1^ [Amide III (β-sheet, coil)] and 1,450 cm^−1^ (CH_2_ bending vibration) that had been verified previously in the literature (Huang et al., 2021). 838 cm^−1^ peak was selected as a representative of the virus in the mapping experiments. In addition to TA and the virus, also the distribution of CS was monitored. For CS, a representative fingerprint, 2,890 cm^−1^, was found, which did not interfere with the peaks of others.

Using Raman mapping, the distribution of used materials in 65 μm × 65 μm areas was observed. TA and CS showed several areas in the monitored functionalized paperboard (P 15) with positive signal of them (Figure 9B). Both were observed in similar areas but, interestingly, they were not totally colocalizing. Moreover, HCoV-OC43, deposited on the paperboard with 1% TA functionalization, was found only in the areas with relatively high amount of TA following the sample flushing (Figure 9B). The Raman mapping also indicated that the amount of HCoV-OC43 in the CS concentrated regions is insignificant.

## 4 Discussion

As current and future pandemics and epidemics pose a serious threat to the society and health care systems, there is an urgent need for new ways to reduce viral load in the environment and on different surfaces. During Covid-19 pandemic is was recognized that packaging industry requires new alternative options for the future to replace extensive usage of sanitizers (Gopal and Muthu, 2023). As cellulose is one of the most prevalent and broadly employed polymers in the packaging industry besides plastic (Liu et al., 2021) and several viruses can remain infectious on top of it for long periods of time, there is a need for new cellulose-based antiviral applications. Bio-based functionalized surfaces to fight against microbes are still rather rare, but they can provide an efficient, safe, and environmentally friendly option to inactivate microbes and prevent further infections. Hence, it was studied if commercially available TA and tannin-rich spruce bark and willow extracts could be bound to different cellulose-based materials with selected binding chemistries and prevent infectivity of both non-enveloped and enveloped viruses. The antiviral coating could be utilized in numerous product and food packaging materials and hygiene products.

TA is a mixture of polyphenolic compounds. These compounds most commonly consist of a central glucose unit esterified with varying number of gallic acid molecules forming a mixture of galloylglucoses and gallotannins. TA has been shown to have antimutagenic, anti-inflammatory and antitumor activities and TA neutralizes free radicals, thus making this mixture of compounds a very promising tool for numerous health applications (Kaczmarek, 2020; Jing et al., 2022).

The results of this study demonstrated that TA in solution exhibited excellent antiviral activity at low amounts against both non-enveloped enteroviruses as well as enveloped coronaviruses, both seasonal and SARS coronaviruses. This was not unexpected as TA has been shown to carry antiviral activity against several viruses (Kaczmarek, 2020), including enveloped virus influenza A (Theisen et al., 2014) and non-enveloped viruses norovirus (Zhang et al., 2012) and hepatitis C (Shirasago et al., 2019). Theisen et al. (2014) further showed that in addition to purified TA, the bark extracts from *Hamamelis Virginiana* L. showed antiviral efficacy in the similar μM levels as the commercial TA. TA has also been considered as a potential antiviral against SARS-CoV-2 as it interferes with receptor binding (Wang S.-C. et al., 2020; Haddad et al., 2022). In addition, some hydrolysable metabolites of TA are recognized as potential antivirals inhibiting SARS-CoV-2 protease activity (Li et al., 2021). The antiviral efficacy has been associated with the complex large TA whereas much lower activities have been found with smaller tannin structures pentagalloyl glucoside or gallic acid (Theisen et al., 2014).

Some earlier studies have demonstrated a possibility to utilize TA in preparation of antiviral functionalized materials. In a study by Kim et al. (2021) TA-coated HEPA filters showed considerable efficiency against influenza A by displaying capture performance of up to 2,730 PFU/mm^2^ within 10 min. Also, TA containing nanoparticles have been shown to induce antiviral activity (Orłowski et al., 2018). Furthermore, in our previous study, TA also showed efficacy against CVB3 when it was bound to handsheets using a dip coating method without any additional binding polymers or chemicals (Jyske et al., 2023). Although in that study, TA was not strongly bound to the surface and probably efficacy was based on easily leaching TA from the surface, TA showed its promise also against the stable enteroviruses.

Our results here indicated that TA showed great potential against non-enveloped CVB3 on functionalized cellulose-based paperboard and SC material. Full inhibition of CVB3 infectivity was detected when only 0.1 or 0.5% TA solution (depending on binding chemistry) was utilized in sample preparation. Significantly, the effect was observed after a very short, 5-min incubation at RT on top of samples. The antiviral effect of bio-based functionalized materials has been rarely displayed after a very short contact time between virus and surface. TA functionalization showed promising potential also against HCoV-OC43 as already 0.1% TA treated paperboard was effective against HCoV-OC43 when the viral amount was moderately high (9.0 × 10^5^ PFU/ml). When the viral load was increased to even higher amount (9.0 × 10^6^ PFU/ml) the antiviral effect on TA-containing paperboard was still moderate. The viral titre of coronaviruses has usually varied from 10^3^ PFU/ml up when their persistence has been tested on inanimate surfaces (Kampf et al., 2020). Thus, the inoculum of 10^5^ to 10^6^ in our experiments is relatively high.

We have previously shown that, besides TA, various wood extracts have great potential to lower virus infectivity. Willow bark-based extracts exhibited antiviral efficacy against enterovirus Coxsackievirus A9 in solution already after 45-s contact time with the virus in solution (Tienaho et al., 2021). Also, Norway spruce bark extracts were able to totally block the infectivity of Coxsackievirus A9 (CVA9) in solution earlier (Pap et al., 2021) and now against CVB3 also in this current study. However, in this study the antiviral efficacy of spruce bark and willow bark/biomass extracts against enteroviruses could not be retained when bound to paperboard. This is probably a result of an inadequate amount to be accessible when using various polymers to bind the antiviral to the surface. Antiviral action was likely masked by other components of the solution such as chitosan. Also, the concentration used for functionalization could have been too low for gaining antiviral efficacy. In our previous study (Jyske et al., 2023), the paper material was dipped into TA or bark extract containing solution (10× more concentrated than here) which likely left the antiviral moieties more easily accessible to the viruses. As no binding polymers were used, there was no covalent bonding between TA and surface fibers, and consequently TA could have detached more easily from the surface. However, here the aim was to gain stronger binding with smaller amount of antivirals to the surface, which then resulted in poorer outcome for the bark extracts. In contrast, TA showed great efficacy suggesting that the binding polymers were not blocking all active moieties in TA. In the future, the bark extracts could be purified from potential carbohydrate contaminations to yield higher binding to the surfaces and higher virucidal activities.

The immobilization method used in this *t* study rests on chemical bonding between material and antivirals crosslinked with different polymers. CS, C-PVAm, SMA and C-PAM were utilized in this study to conjugate TA and bark extracts into cellulose materials. The formation of non-covalent and covalent bonds between functional groups of antiviral and surface materials are expected to lengthen the shelf life of antiviral efficacy. The experiments were performed over the span of 2 years, and the antiviral efficacy of the samples was confirmed to be great still after 2 years. Importantly, immobilization reduces the leakage of antivirals from the material (Fu and Dudley, 2021), which is crucial for several practical applications concerning safety. If antiviral functionalization is aimed to be utilized in, e.g., food packaging, it is important that the possible consumer ingestion of antivirals is being restricted. Other methods used regularly for surface coating include for example dip coating, electrospinning, spraying, bar coating, layer by layer coating and *ex situ* coating (Fu and Dudley, 2021).The used chemistry likely contributes strongly to the possible covalent binding of antivirals.

The results indicated that all different tested binding polymers served well as antiviral effect was achieved when they were utilized in the coatings. However, none of them were remarkably superior to others: replacement of CS with C-PVAm or switching between SMA and C-PAM did not remarkably affect the antiviral activity. Furthermore, the results showed that TA was the most crucial component for achieving antiviral effect against CVB3 on TA functionalized materials. The loss of antiviral effect was detected if TA concentration was low enough. Also, CVB3 remained fully infectious when TA was replaced with bark extracts for functionalization but still contained other components, including chitosan. However, the possible contributing factor of CS and other polymers used for binding the antivirals cannot be totally ruled out. CS is an organic polysaccharide and a deacetylated derivative of chitin has gained interest in antimicrobial design. Antiviral properties have been demonstrated against plant viruses (Jia et al., 2016; Abdelkhalek et al., 2021) and animal and human infecting viruses (Zheng et al., 2016; Jaber et al., 2022; Mahboub et al., 2022). Besides antimicrobial properties CS is considered as relatively non-toxic, biodegradable, and biocompatible (Kean and Thanou, 2010). CS based nanoparticles for antiviral drug delivery and vaccination have lately drawn interest as CS nanoparticles can be administered by transmucosal routes (Boroumand et al., 2021). In our study the CS was first radicalized to enable its conjugation with TA and bark extracts. Hydroxyl and amine groups in the structure of CS permit chemical modification to deliver better properties in terms of antimicrobial efficacy (Tan et al., 2022; Umar et al., 2022). It has also been demonstrated that there is attractive interaction between CS and cellulose possibly caused by bridging between smooth CS surface and cellulose chains (Holmberg et al., 1997).

Polyvinylamines like C-PVAm utilized in this study have been previously successfully utilized to bind biologically active agents; to anchor photoactive dyes into filter paper to gain bactericidal effect (Brovko et al., 2009) and to immobilize bacteriophages on cellulose membranes to reduce the growth of bacteria (Anany et al., 2011). Similarly, SMA has been exploited in packaging industry to bind bioactive compounds. SMA can be employed to prepare functional polymers as its succinic anhydride units can be coupled to active compounds (Jeong et al., 2002). Polycations such as C-PAM exploited in this study are also promising in the field of antimicrobial design as they can be linked with antimicrobial agents that can prevent bacterial growth and inhibit viral infectivity (Xue et al., 2014). Citric acid used in our binding chemistry here, has been applied commonly in combination with cellulose-based materials to improve some properties of the products, involving flexibility and compressive strength (Priya et al., 2014; Widsten et al., 2014) Citric acid has also shown some antibacterial (Kundukad et al., 2020) and antiviral activities on surfaces (Lombardi et al., 2008), and it has been utilized to cross-link chitosan (Zhuang et al., 2020). The nature of chemical bonding was not confirmed in our study but will be the focus in future studies.

In this study, CVB3 remained infectious on top of all three cellulose-based reference materials after short 5-min incubation whereas HCoV-OC43 lost some infectivity on top of reference PC and SC materials. Generally, it is known that coronaviruses are more stable on impermeable surfaces than on porous surfaces (Chatterjee et al., 2021). Enteroviruses have a stable capsid structure composed of proteins as their outer layer. On the other hand, coronaviruses have a lipid envelope that is more susceptible to breakages and drying that could consequently more easily lead to the loss of viral infectivity. Cellulose itself is a hydrophilic polymer by nature as it has several hydroxyl groups (-OH) that allow it to form hydrogen bonds with water molecules (Klemm et al., 2005). This hydrophilic nature of cellulose thus quickly leads to absorption of virus containing liquid on the cellulose-based surface bringing virus into close contact with the material.

Contact angle measurement revealed that SC was the most hydrophilic material in the study absorbing the water droplet immediately. Thus, it was not surprising that the viral infectivity of HCoV-OC43 was already a bit reduced on the reference material. However, as the infectivity of coronavirus was also reduced on the most hydrophobic PC material, the inhibitory effect in this case cannot be directly linked with hydrophilicity/hydrophobicity.

Previous studies have suggested that coronaviruses and enteroviruses can remain infectious from minutes to several days on porous surfaces depending on virus strain and infectious dose. Remarkably, also various SARS-CoV strains were found active on cardboard until 24 h (van Doremalen et al., 2020) and on top of paper even after 4–5 days (Duan et al., 2003). Lai et al. (2005) demonstrated that stability of SARS-CoV (Strain GVU6109) on paper varied depending on the viral titre; the virus was inactivated in less than 5 min with 10^4^ TCID50/ml and in 3 h with 10^5^ TCID50/ml. In our study, the infectivity of HCoV-OC43 was remarkably reduced on the functionalized paperboard after treating 10^5^ TCID50/ml amount of virus for 5 min. On reference paperboard HCoV-OC43 was infectious following 5-min treatment, but after 24-h incubation no infectious virus was recovered. Stability of enteroviruses on surfaces has been studied less compared to coronaviruses. Abad et al. (1994) demonstrated that poliovirus infectivity persisted up to 30 days on paper with only 1.5 reduction in viral titre (log10 Nt/N0) when virus was suspended in PBS. The viral infectivity of poliovirus was retained better at 4°C with 90% relative humidity than at 20°C with 50 and 85% relative humidity. In our study no loss of CVB3 infectivity was observed on reference materials during 5-min incubation, but after 24-h treatment viral infectivity was fully inhibited Importantly, efficient loss of enterovirus infectivity was demonstrated as a result of TA functionalization after a short period of time.

It could be deduced from the Raman spectroscopy mapping, that after flushing the virus from TA functionalized paperboard most of the remaining HCoV-OC43 was still bound to the TA containing areas on functionalized paperboard. This implies that some interaction or binding is likely to exist between the virus and TA in the material already after short 5-min treatment. Surprisingly, according to qPCR results there was more RNA of HCoV-OC43 bound to the reference paperboard than to the functionalized material after flushing the samples. There could be several factors explaining the result. The functionalizing treatment might alter the properties of paperboard by making it easier for HCoV-OC43 to detach due to flushing. Although most of the viral RNA easily detached from the functionalized material by flushing the surface, the short-lived binding to the TA-functionalized surface had changed the viruses as they had lost infectivity to a large extent.

In contrast to coronaviruses, qPCR results with CVB3 demonstrated that most enteroviruses remained bound to the TA functionalized paperboard. The inhibition of CVB3 infectivity after treatment on functionalized cellulose could be then widely caused by the strong viral binding ability of the functionalized paperboard. Interestingly, it was observed that paperboard material was more hydrophilic following the functionalization with 1% TA. This feature could also more easily bring the virus to a closer contact with active components. The functionalized PC material showed little efficacy against CVB3, and the material was still strongly hydrophobic after functionalization, further explaining the low efficacy of PC material. Optical density measurements indicated that only a very small amount of TA was leaking from the paperboard during 5-min flushing. Thus, the usage of chitosan and other binding polymers immobilized TA efficiently and leaching of TA was mainly prevented. However, as the EC50 value of TA against CVB3 was remarkably low, even small amounts of released TA could theoretically have antiviral efficacy. But, as samples like PC 15 and 16 showed somewhat higher leaching, and there was no remarkable antiviral efficacy detected on these PC materials, it could be deduced that the amount of TA leached from the materials was not high enough to cause the antiviral efficacy.

Tannins, including TA, are known to be able to bind and precipitate proteins as albumin and other plasma proteins (Engström et al., 2019; Pinto et al., 2019). The protein binding ability of TA lies behind formation of hydrogen bonds and hydrophobic interactions between TA and proteins. Hydrophobic interaction arises between aromatic rings of TA and hydrophobic side chains of amino acids in proteins, whereas hydrogen bonds can form between phenolic hydroxyl group of TA and carboxyl groups of proteins (Jafari et al., 2022). It was denoted in our previous study that polyphenols like epigallocatechin gallate and resveratrol can cluster and stabilize enteroviruses by binding to the virus surface, and thus prevent the viral entry into cells (Reshamwala et al., 2021). Hence, the antiviral mechanism of TA could be also related to the ability of some polyphenols to bind to viruses.

In conclusion, it was demonstrated here that TA and spruce bark extract exhibit broadly acting antiviral activity against non-enveloped CBV3 and enveloped HCoV-OC43 and SARS-CoV-2. Furthermore, TA can be exploited to prepare antiviral coating on cellulose-based materials utilizing different binding polymers with excellent antiviral efficacy against CVB3 and HCoV-OC43. The produced antiviral surfaces thus show promise for future use to increase biosafety and biosecurity by reducing pathogen persistence. Produced antiviral coatings could be utilized widely in cellulose-based packaging materials and products. Suitability of the coating method for antiviral functionalization of textiles, including masks, personal protective equipment and other textiles used in hospitals and public transportation could be also assessed in the future.

## Data availability statement

The original contributions presented in the study are included in the article/supplementary materials, further inquiries can be directed to the corresponding author.

## Author contributions

MH: Conceptualization, Data curation, Formal Analysis, Investigation, Writing – original draft. AE: Data curation, Investigation, Methodology, Writing – original draft. DR: Data curation, Writing – original draft. ML: Methodology, Writing – review & editing. JT: Conceptualization, Project administration, Writing – original draft, Writing – review & editing. PK: Resources, Writing – original draft. JL: Conceptualization, Data curation, Methodology, Writing – review & editing. TJ: Conceptualization, Funding acquisition, Methodology, Supervision, Writing – review & editing. MP: Conceptualization, Methodology, Supervision, Writing – review & editing. VM: Conceptualization, Funding acquisition, Methodology, Supervision, Writing – review & editing.
